# Genetic Studies of a Cluster of Acute Lymphoblastic Leukemia Cases in Churchill County, Nevada

**DOI:** 10.1289/ehp.9025

**Published:** 2006-11-30

**Authors:** Karen K. Steinberg, Mary V. Relling, Margaret L. Gallagher, Christopher N. Greene, Carol S. Rubin, Deborah French, Adrianne K. Holmes, William L. Carroll, Deborah A. Koontz, Eric J. Sampson, Glen A. Satten

**Affiliations:** 1 Division of Laboratory Sciences, National Center for Environmental Health, Centers for Disease Control and Prevention, Atlanta, Georgia, USA; 2 Pharmaceutical Sciences, St. Jude Children’s Research Hospital, Memphis, Tennessee, USA; 3 Division of Environmental Hazards and Health Effects, National Center for Environmental Health, Centers for Disease Control and Prevention, Atlanta, Georgia, USA; 4 Hassenfeld Children’s Center, New York University School of Medicine, New York, New York, USA

**Keywords:** arsenic, children, cluster, DDE, enzymes, genetics, leukemia, polymorphism, tungsten

## Abstract

**Objective:**

In a study to identify exposures associated with 15 cases of childhood leukemia, we found levels of tungsten, arsenic, and dichlorodiphenyldichloroethylene in participants to be higher than mean values reported in the National Report on Human Exposure to Environmental Chemicals. Because case and comparison families had similar levels of these contaminants, we conducted genetic studies to identify gene polymorphisms that might have made case children more susceptible than comparison children to effects of the exposures.

**Design:**

We compared case with comparison children to determine whether differences existed in the frequency of polymorphic genes, including genes that code for enzymes in the folate and purine pathways. We also included discovery of polymorphic forms of genes that code for enzymes that are inhibited by tungsten: xanthine dehydrogenase, sulfite oxidase (*SUOX* gene), and aldehyde oxidase.

**Participants:**

Eleven case children were age- and sex-matched with 42 community comparison children for genetic analyses. Twenty parents of case children also contributed to the analyses.

**Results:**

One bilalleleic gene locus in *SUOX* was significantly associated with either case or comparison status, depending on which alleles the child carried (without adjusting for multiple comparisons).

**Conclusions:**

Although genetic studies did not provide evidence that a common agent or genetic susceptibility factor caused the leukemias, the association between a *SUOX* gene locus and disease status in the presence of high tungsten and arsenic levels warrants further investigation.

**Relevance:**

Although analyses of community clusters of cancer have rarely identified causes, these findings have generated hypotheses to be tested in subsequent studies.

The Nevada State Health Division (NSHD), with assistance from the Centers for Disease Control and Prevention (CDC), conducted a study to identify environmental exposures in the Churchill County community where 15 cases of childhood leukemia,14 of which were acute lymphoblastic leukemia (ALL), had been diagnosed between 1997 and 2002. State health officials estimated that fewer than 2 cases would have been expected (Steinmaus et al. 2004). The detailed methods, results, and recommendations of this investigation have been documented by Rubin et al. (2007) in this mini-monograph.

Although the investigation found higher levels of potentially toxic substances, including tungsten, arsenic, and the dichlorodiphenyl-trichloroethane (DDT) metabolite dichloro-diphenyldichloroethylene (DDE) in residents of Churchill County than the values reported in the National Report on Human Exposure to Environmental Chemicals (CDC 2003b), case and comparison families had similar body burdens of these contaminants. Because the two groups had similar concentrations, CDC, in collaboration with Mary Relling of St. Jude Children’s Research Hospital and with the advice of William Carroll, Head of the Children’s Oncology Group (COG) Acute Leukemia Disease Committee, conducted genetic studies in an effort to identify gene variants that might have made case children more susceptible than comparison children to adverse effects of the exposures documented in Churchill County. As a result of the high levels of tungsten found in residents of Churchill County, the investigation also included a study to identify variant forms of three genes that code for enzymes that are inhibited by tungsten and may, as a result, affect DNA synthesis, hematopoiesis, or detoxification of contaminants. For a review of current knowledge of tungsten, including environmental chemistry, toxicologic properties, and ongoing investigation into its possible toxicity, see Koutsospyros et al. (2006).

We also attempted to determine whether the frequency and types of cytogenetic aberrations found in bone marrow specimens from case children were consistent with the frequency of these abnormalities found in the general population of children with ALL enrolled in COG protocols or whether differences in these aberrations provided information that would suggest a common environmental exposure. The following is a report of results of genetic studies conducted on specimens in the Churchill County case–control study.

## Materials and Methods

### Study participants

We enrolled a total of 205 participants representing 14 (of the 15 eligible) case families and 55 comparison families and asked them to complete mailed questionnaires, participate in personal interviews, and donate biological specimens. We excluded 1 of the 14 cases from the analysis because the child lived in the target area for only a short time before diagnosis. The family of another child declined to participate. A third case was excluded because the diagnosis was acute myelogenous leukemia rather than ALL, leaving 11 case children for our analyses. Community-based reference children were matched to case children on the basis of year of birth and sex. The original goal to match four community-based reference children to each case child was not always achieved. Data from 42 comparison children matched to these 11 case children are also included in our analyses. Twenty parents of case children also provided genetic data. Data from the 2 additional cases excluded from our analysis (because of residency and diagnosis) and the 6 control children matched to them were included in single nucleotide polymorphism (SNP, pronounced “snip”) discovery. Blood and cheek-swab specimens were collected for DNA extraction. The study protocol and procedures for written informed consent and assent were reviewed and approved by the CDC Institutional Review Board (IRB; protocol 3195; CDC 2003a) and complied with all applicable requirements of the U.S. regulations. All adult participants signed IRB-approved, written informed consent. Children 7 years of age and older signed written assents, and the parents or legal guardians of children younger than 7 years signed written consents.

### Genetic studies

Two types of genetic information were assessed in this case-comparison study: *a*) We collected and assessed all available information about the gene or chromosomal abnormalities characteristic of a child’s leukemia cells that were ascertained at case presentation from bone marrow specimens and used for diagnosis and classification of leukemia. These abnormalities include chromosomal aberrations such as translocations and abnormal numbers of chromosomes (e.g., hyperdiploidy) and gene mutations (Table 1). *b*) We also assessed inherited genetic variation that may affect susceptibility to leukemia in people exposed to environmental leukemogenic agents (Pui et al. 2004; Smith et al. 2005). This variation includes polymorphisms in genes that code for proteins involved in metabolism of drugs and environmental toxicants, in synthesis of DNA, in innate immunity, and other enzymes. The most abundant type of polymorphism is a SNP. A locus refers to a particular base pair in the genome. If there is genetic variability at a locus, each variant is referred to as an allele. A person inherits two alleles, one from each parent, at each autosomal locus. All loci in this study were bi-allelic; the majority were SNPs, although one was a deletion and one was a variable number of tandem repeats polymorphism (VNTR) with two alleles.

We genotyped case and comparison families for genes that code for proteins that may affect, or be affected by, elevated concentrations of tungsten, arsenic, pesticide metabolites, or other environmental agents. All gene names, gene symbols, and their accession numbers are from GenBank (http://www.ncbi.nlm.nih.gov/Genbank/). The 13 genes that we focused on include genes in the folate/purine biosynthesis pathway: methylene tetrahydrofolate reductase (*MTHFR,* GenBank accession no. AY338232), serine hydroxymethyltransferase 1 (*SHMT1*; GenBank accession no. AK223552), reduced folate carrier (*SLC19A1*; GenBank accession no. BC003068), thiopurine *S*-methyltransferase (*TPMT*; GenBank accession no. BC009596), and thymidylate synthetase (*TYMS*; GenBank accession no. BC083512). We also examined genes involved in xenobiotic metabolism including ATP-binding cassette, subfamily B (*ABCB1*; GenBank accession no. *AY910577*), glutathione *S*-transferase pi (*GSTP1*; GenBank accession no. BT019950), and NAD(P)H dehydrogenase, quinone 1 (*NQO1*; GenBank accession no. BC007659). In addition, we genotyped polymorphisms in the vitamin D receptor gene (*VDR*; GenBank accession no. AB002168), which plays a role in cell differentiation, and the gene coding for mannose-binding lectin (protein C) 2 (*MBL2*; GenBank accession no. BC096181), an important mediator component of the innate immune defense system.

We also performed SNP discovery in three genes that code for enzymes inhibited by tungsten [xanthine dehydrogenase (XDH; EC1.1.1.204, National Center for Biotechnology Information (NCBI) geneID: 7498, GenBank accession no. NM_000379.3), aldehyde oxidase (AOX1; EC1.2.3.11; probably identical to retinaldehyde oxidase, EC1.2.3.1, NCBI geneID: 316, GenBank accession no. NM_001159.3), and sulfite oxidase (SUOX; EC1.8.3.1, NCBI geneID: 6821, GenBank accession no. NM_000456.2)], using specimens from 11 case children and 24 matched comparison children. The comparison children were selected by randomly sampling two (or in two instances, three) of the comparison children matched to each case child. In addition, to characterize variation in these genes as a resource for future studies, we also included in our SNP discovery 52 specimens from the Human Variation Panels of the Human Genetic Cell Repository (sponsored by the National Institute of General Medical Sciences and deposited with the Coriell Institute for Medical Research). Briefly, amplification primers were designed, and an amplicon tiling model spanning both coding and regulatory regions was developed. Polymerase chain reaction (PCR) amplicons covering all intron/exon borders, full exon sequences, known promoter sequences, and 3′ untranslated regions (UTRs) of each of the three genes were amplified, and amplicons were sequenced bidirectionally using Big Dye version 3.1 (Applied Biosystems, Foster City, CA) terminator chemistry in conjunction with ABI 3700/3730xl DNA analyzers (Applied Biosystems). For these three genes, we used computer modeling with the freely available programs PolyPhen (http://genetics.bwh.harvard.edu/pph/; Ramensky et al. 2002) and SIFT (sorting intolerant from tolerant) (http://blocks.fhcrc.org/sift/SIFT.html; Ng and Henikoff 2001) to predict which SNPs would cause functional changes in the proteins for which the genes coded.

The polymorphisms and their dbSNP (Single Nucleotide Polymorphism database; http://www.ncbi.nlm.nih.gov/SNP/) identifier, if available, are listed below. Polymorphisms in *TYMS* 28-bp tandem repeat and *TPMT* variants 460G→A (rs1800460) and 719A→G (rs28933403) were genotyped as described by Relling et al. (2004). We genotyped *SLC19A1* 80A→G polymorphism (rs1051266) by direct sequencing, using primers AGTGTCAC CTTCGTCCCCTC (forward and sequencing) and CTCCCGCGTGAAGTTCTT (reverse). We genotyped the *SHMT1* 1420C→T polymorphism (rs1979277) by a modification of a Taqman assay (http://snp500cancer.nci.nih.gov; SNP500 assay number 003_1859), using amplification primers ATTTGTGAAGAAAACATGAA AAA (forward) and AGACTGGCAGGG GATAAGTA (reverse). The *GSTP1* 313A→G (rs1695)*, NQO1* 609C→T (rs1800566), *MBL2* codon 54A→G (rs1800450), *ABCB1* 3435C→T (rs1045642), *MTHFR* 677C→T (rs1801133), and *VDR* start codon FokI polymorphism (rs2228570) were genotyped by DNAPrint Genomics (Sarasota, FL) in a multiplex PCR followed by single base extension.

### Statistical analysis

We performed a (univariate) matched logistic regression analysis using data from each locus listed in Tables 2 and 3. Note that all loci are bi-allelic. For all analyses, we used a co-dominant model that treated the number of minor alleles at the locus of interest as the risk factor. For each locus, the “minor” allele is the less common allele. We calculated point estimates, 95% (exact) confidence intervals (CIs) and (exact) *p*-values using SAS PROCLOGISTIC (version 9.3.1; SAS Institute Inc., Cary, NC). Analyses of *AOX1, SUOX*, and *XDH* included data from 11 case children and 24 comparison children matched with the case children who were included in the SNP discovery. Analyses of all other loci included data from 11 case children and 42 matched comparison children.

For loci in *AOX1, SUOX,* and *XDH,* we also considered variables that summarize the effect of several loci. Specifically, we compared the ratio of minor alleles with the total number of alleles genotyped between case children and comparison participants. A “minor” allele was declared “rare” if there were two or fewer copies of that allele observed in the Churchill County study population, and a locus was called a rare-allele locus if the minor allele was rare. We also compared the ratio of rare alleles with the total number of alleles at rare-allele loci genotyped between case children and comparison participants. Finally, we compared case and comparison participants’ potentially deleterious alleles (as determined by PolyPhen or SIFT). A locus was called a deleterious-allele locus if the minor allele was considered deleterious. We compared the ratio of deleterious alleles with the total number of alleles at the deleterious-allele loci genotyped between case children and comparison participants. For this analysis, we used both the PolyPhen and SIFT determinations of what alleles were deleterious. These summary variables were also analyzed using exact conditional logistic regression for matched sets and were calculated using SAS version 9.1.3.

In addition, for *AOX1, SUOX* and *XDH*, we compared the distribution of groups of variants that are inherited together on the same chromosome (haplotypes). When constructing haplotypes, we considered only loci for which the minor allele was not rare. For *AOX1*, the 6-locus haplotypes using loci 78499A→G, 86558A→G (rs3731722), 87950T→G, 88099G→A (rs1050887), 88169A→delA, and 88248G→T were thus considered. For *SUOX* we considered a 2-locus haplotype using loci –628G→A (rs7297662) and −586T→A (rs773126). We did not conduct haplotype analyses for *XDH*, as all loci had rare minor alleles. The common gene variant is denoted as “0” and the minor variant as “1”. For example, for *SUOX*, the haplotype 10 corresponds to having the minor variant at locus –628, A, and the common variant at locus –586, T. For each gene, we tested the association between haplotypes and disease status assuming a co-dominant model for each haplotype with frequency > 5% in the study population, using the robust score test in CHAPLIN (Epstein and Satten 2003; Satten and Epstein 2004; see http://www.genetics.emory.edu/labs/epstein/software/chaplin/index.html). For each gene we also calculated the (marginal) effect of each haplotype with frequency > 5% on the odds of disease. For these univariate analyses, we considered co-dominant, dominant, and recessive models. We determined genotypes of available parents of case children for each gene listed in Table 3. We conducted transmission-disequilibrium tests (TDTs) for linkage and association. To include data from two case children with only one available parent, we used the 1-TDT (Sun et al. 1999).

## Results

Case children (7 boys and 4 girls) ranged in age from 2 to 19 years at diagnosis and from 3 to 20 years at time of sample collection. Characteristics of the leukemias of 11 case children on which specimens were received are presented in Table 1. The distribution of immunophenotypes (T-cell vs. B-cell leukemia determined by flow cytometry) among case children was similar to what is seen in the general population of children with ALL in the United States (COG, personal communication). The TEL-AML1 translocation t(12;21), found in about 25% of patients with ALL (Romana et al. 1995), was identified in the cells of two children between the ages of 2 and 10 years. Hyperdiploidy, another cytogenetic aberration common in ALL, was found in cells from a third child between the ages of 2 and 10 years. In all three instances, chromosomal aberrations were found in association with precursor B-cell ALL cases. The frequency of cytogenetic chromosomal aberrations, such as translocations and hyperdiploidy in this group of leukemia cases, could not be compared with the frequency of these aberrations found in the general population of children with ALL because, in most cases, bone marrow was not stored, testing was not done, or cells did not divide for metaphase analysis.

Polymorphism discovery by DNA sequencing was performed to detect variant forms of genes that code for enzymes inhibited by tungsten. A total of 125 variations in *AOX1*, 29 variations in *SUOX*, and 124 variations in *XDH* were identified, of which 8 *AOX1* loci, 7 *SUOX* loci, and 5 *XDH* loci were polymorphic in our study population. Analysis was restricted to polymorphisms in the coding regions of the genes (cSNPs) that resulted in amino acid substitutions or those in potential regulatory regions, including the promoter, exon/intron boundaries, and 3′ UTR (Table 2). Minor allele frequencies for all loci found to be polymorphic in our study population are listed in Tables 2 and 3.

We found no differences in frequencies of minor alleles between case and comparison participants for 10 genes that code for proteins involved in the folate/purine biosynthesis pathway, in xenobiotic metabolism, in cell differentiation, or in innate immunity (Table 3).

Univariate logistic regression (Tables 4 and 5) identified only one locus (*SUOX* –628G→A) that was significantly associated with disease status (*p =* 0.007). All 11 case children had at least one copy of the minor allele (A); no child carrying a GG genotype had leukemia. Fifty-five percent of the case children and 25% of the comparison children were homozygous for the minor allele (A). Forty-six percent of the comparison children were homozygous for the G allele. The proportion of minor *SUOX* alleles also differed significantly between case and comparison children (*p* = 0.001); this proportion was not significantly different for *AOX1* or *XDH*. The proportion of rare gene variants was borderline significantly different between case and comparison children for *AOX*1 (mid-*p*-value for exact score was 0.06); this is because 2 of the 11 case children, but none of 24 comparison children, had a rare *AOX1* allele. No significant differences between rare allele frequencies in case and comparison children were found for *XDH* or *SUOX*. Further, the proportion of deleterious alleles, as determined by the PolyPhen and SIFT programs, was not significantly different between case and comparison children (mid-*p*-values for exact score test were 0.58 for PolyPhen and 0.66 for SIFT). Finally, the TDT revealed no locus in linkage or association with a trait locus (Table 6).

### Aldehyde oxidase and sulfite oxidase haplo-type analyses

The haplotypes estimated to have non-zero frequency in the *AOX1* and *SUOX* genes calculated using the expectation–maximization (EM) algorithm are given in Table 7. Using the robust score test for haplo-type effect, *AOX1* had *p* = 0.38, based on 3 degrees of freedom (*df* ), and *SUOX* had *p* = 0.05, based on 3 *df*. We also examined individual haplotype models for each haplotype with a frequency of at least 5% in the study population. For each haplotype, we compared the null hypothesis that no haplotypes affect the odds of disease with the alternative hypothesis that the selected haplotype affects the odds of disease. Note that results for the recessive model may be unreliable because of the small number of children having two copies of the risk haplotype. Further, although *AOX1* haplo-type 100000 (G–A–T–G–A–G) also has a nominally significant *p*-value under a recessive model, it has a protective effect. *SUOX* haplo-type 10 (A–T) has a significant *p-*value for both co-dominant and dominant models; the first position corresponds to the only locus significantly associated with case status in univariate analyses (Table 4). *SUOX* haplotype 00 (A–T) is close to borderline significant but protective for the co-dominant and recessive models. In general, the sign of the haplotype effect in *SUOX* tracks the genotype at the first locus, suggesting this locus alone accounts for the observed association.

## Discussion

Investigators understood from the outset that finding a cause of leukemia in this study was unlikely because of the small number of case children. Nonetheless, we decided to perform genetic testing because the results from this study may be useful in generating hypotheses for subsequent leukemia clusters and because of the possibility of performing meta-analysis on data from this and subsequent studies to elucidate causal mechanisms.

We studied two types of genetic information: *a*) the cytogenetic and molecular changes that are found in bone marrow cells from ALL case children at diagnosis and *b*) the normal genetic variations that occur in people that might make some more susceptible than others to the effects of environmental chemicals.

With regard to cytogenetic studies, no evidence was found to suggest that a common environmental exposure caused the leukemias, although this possibility could not be ruled out. Cytogenetic studies on pretreatment bone marrow specimens suggested that the chromosomal aberrations and cell immunophenotypes were present in approximately the same proportion as in the general population of childhood ALL cases in the United States*,* although this could not be confirmed for chromosomal aberrations because testing was not performed in many cases and bone marrow was not stored for later testing.

For normal variation, we compared the distributions of gene variants in case and comparison families for genes that code for four groups of proteins: enzymes that are inhibited by tungsten, folate pathway enzymes, proteins involved in cell differentiation, and enzymes that activate or detoxify carcinogens. We also studied a gene variant in a protein involved in innate immunity.

Three mammalian enzymes—xanthine dehydrogenase, sulfite oxidase, and aldehyde oxidase—require molybdenum as a cofactor (Garner and Stewart 2002). Tungsten readily replaces molybdenum as a cofactor in these enzymes, thus inactivating them (L’vov et al. 2002). Tungsten has also been shown to prevent incorporation of molybdenum without itself being incorporated into xanthine dehydrogenase. Sulfite oxidase has also been shown to be inhibited by arsenite *in vitro* (Gardlik and Rajagopalan 1991). Although it is not clear how inhibition of these enzymes might affect risk for leukemia, plausible mechanisms can be hypothesized. For example, aldehyde oxidase (probably identical to retinaldehyde oxidase) irreversibly converts retinaldehyde to retinoic acid, which mediates hematopoiesis and progenitor cell differentiation through its effects on *Hox* gene expression during embryogenesis (Look 1997; Tocci et al. 1996).

A comprehensive survey of the polymorphisms found in the coding and regulatory regions of these three genes revealed only one locus (*SUOX* –628) was significantly associated with disease status. If we adjusted for the number of comparisons made in the univariate logistic regression analyses, this locus would no longer be significant. However, the pattern of genotypes is noteworthy (all children with leukemia carried at least one copy of the A allele; no child having the GG genotype had leukemia).

Sulfite oxidase is required for the physiologically essential oxidation of sulfite to sulfate, the final step in the oxidative breakdown of the amino acids cysteine and methionine. Deficiency of sulfite oxidase due to mutations in the human *SUOX* gene causes severe neurologic deficits resulting in death at an early age or mental retardation [for a review, see Tan et al. (2005)].

We found nothing in the scientific literature about the effect of the SUOX –628G→A variant on sulfite oxidase activity. This polymorphism is in the 5′ UTR, between untranslated exons and the first translated exon. Although the physiologic consequences of polymorphisms in untranlsated regions remain to be fully elucidated, these polymorphisms may regulate gene expression through effects on mRNA stability, localization, and translational efficiency (Gebauer and Hentze 2004). If this variant were to express less sulfite oxidase protein or code for an enzyme with decreased sulfite oxidase activity, it is possible that exposure to tungsten, arsenic, or both could further decrease activity, thus creating a situation wherein people are vulnerable to adverse effects of decreased sulfite oxidase activity. Many of the comparison children living in Churchill County had this gene variant as well as tungsten and arsenic exposure but did not develop leukemia. For this reason, we think that exposure to additional chemicals would be required to cause adverse health effects.

Whether and how the *SUOX* –628 locus genotype in the 5′ UTR might affect the availability of sulfite oxidase need to be studied further. At this point, we have no evidence to suggest that any genotype at this locus of *SUOX* affects the concentration or activity of sulfite oxidase. Population stratification (case and comparison groups coming from genetically diverse populations thereby showing spurious associations) probably does not explain the finding of an association at the –628 locus with disease status because 95% of case and comparison children identified as white, with the two groups having similar ethnicity. Because of the large number of comparisons made, there are several possible explanations for these associations. They may be because of chance alone; alternatively, carrying the GG genotype may be protective or carrying an A allele may increase risk. Last, the locus we found to be associated with disease status may be in linkage disequilibrium with another unrelated locus. Although the finding of an association is interesting and warrants further research, the genotype at the *SUOX* –628 locus is currently not predictive of any clinical outcome nor does it have any known use in clinical care.

Regarding tungsten mutagenicity, in a study by Miller et al. (2001), exposure of a nontumorigenic, human osteoblast-like cell line to heavy metal–tungsten alloys, dense heavy metal composite materials comprising tungsten (91–93%), nickel (3–5%), and either cobalt or iron (2–4%) resulted in a significant increase in transformation frequency (8.9- ± 0.93-fold). The mechanism for transformation appeared to be increased DNA breakage or chromosomal aberrations. However, tungsten alone did not cause DNA breaks in these cells.

Although the National Toxicology Program (NTP) found no evidence from a literature review that tungsten is carcinogenic (NTP 2003), it is now studying short-, intermediate-, and long-term exposure effects of tungsten using a variety of toxicologic end points including carcinogenicity. Rats and mice of both sexes will be exposed to sodium tungstate dihydrate in their drinking water for periods ranging from 2 weeks to 2 years. Despite the lack of evidence in the literature, the ability of tungsten to inhibit three enzymes that may affect hematopoiesis or detoxification could conceivably alter risk for mutations or chromosomal aberrations.

In previous studies, rats fed high tungsten and molybdenum-free diets were XDH-deficient, which inhibits conversion of purines to uric acid. Because imbalances among deoxynucleotide pools have been linked to mutagenesis, it is plausible that tungsten-induced derangements in purines could be involved in leukemogenesis (James et al. 1994; Kunz and Kohalmi 1991).

Other studies have shown that molybdenum-deficient rats fed tungsten had decreased sulfite oxidase and xanthine oxidase activities. Although these rats appeared healthy, they were more susceptible than control rats to bisulfite toxicity. The lethal dose for intraperitoneal bisulfite was approximately 2.6 times lower for the tungsten-treated animals (Cohen et al. 1973). Sulfite oxidase is also inactivated by arsenite, although by a mechanism different than tungsten inactivation. Arsenite binds to sulfhydryl groups of molybdopterin and in doing so ultimately displaces molybdenum (Gardlik et al. 1991).

Case and comparison specimens were also genotyped for polymorphic forms of genes that code for enzymes in the folate/purine biosynthesis pathway. The folate pathway is essential to single-carbon metabolism, which is critical in DNA synthesis and repair, regulation of gene expression, and protein and polyamine synthesis (Smith et al. 2005). We studied polymorphic forms of folate/purine pathway enzymes including *MTHFR*, *TYMS, SLC19A1*, *TPMT*, and *SHMT1* and found no differences in distribution of polymorphisms between case and comparison families. However, Skibola et al. (1999) found a significant association between two polymorphic forms of *MTHFR,* including the 677C→T polymorphism, and decreased risk for ALL. Skibola et al. (2002) also reported that two other polymorphic genes in the folate pathway conferred decreased risk for ALL: the 5′ UTR polymorphism of *TYMS* and the 1420C→T polymorphism of *SHMT1*. An association between the 677C→T substitution in *MTHFR* and hyperdiploidy, a common cytogenetic aberration in childhood ALL, has also been demonstrated (Pui et al, 2004).

Finally, we genotyped specimens to determine the relative frequencies of polymorphic forms of the gene coding for the carcinogen-metabolizing enzyme *NQO1* (EC1.6.992). *NQO1* protects against oxidative stress by catalyzing the two-electron reduction of quinones to hydroquinones. The 609C→T polymorphism codes for an enzyme with low activity and has been associated with increased risk for ALL in children (relative risk, 1.7; 95% CI, 1.2–2.4) (Krajinovic et al. 2002). Frequencies of the high-risk polymorphism were not significantly different between Churchill County case and comparison children. Our failure to replicate results from previous studies of genetic polymorphisms associated with ALL might be because of the small size of our study population, false positive findings in the original studies (type 1 error), or differences among the genetic make up of the background populations sampled in this and other studies.

Although childhood leukemia has a shorter latency than most solid tumors occurring later in life, exposures that can no longer be documented may have occurred years before the disease was manifested. In the case of leukemia, *in utero* exposures are especially important. Cytogenetic translocations and hyperdiploidy noted in diagnostic bone marrow cells of children with leukemia have been found in the children’s neonatal blood spots, thus indicating that they are induced *in utero* (McHale and Smith 2004). Latency periods have been noted, in some cases, to exceed 10 years. The mechanism for generation of most of the translocations has been suggested to be aberrant repair following abortive apoptosis rather than V(D)J recombination or exposure to topoisomerase II inhibitors (McHale and Smith 2004).

The environmental and genetic factors that we focus on in this article, the *SUOX* –628 locus and exposure to tungsten and arsenic, were present before an increase of incidence of childhood ALL was reported and continued to be present after leukemias were no longer occurring at an elevated rate, thus weakening the case for their having a role in causality. As a result of this investigation, water treatment has been improved and exposure to tungsten and arsenic reduced, although any effects of removing these chemicals have not had time to be manifested.

## Conclusions

From the information obtained in this study and in consultation with members of the COG, limited conclusions can be drawn. The distribution of types of leukemia in Churchill County (for example, the ratio of pre-B-cell to T-cell leukemias and the ratio of standard-risk to high-risk cases) is what one would expect in comparison to the larger population of leukemias studied by the COG in the United States. Although genetic studies did not provide evidence that a common agent or genetic susceptibility factor had caused the leukemias, the association between the *SUOX* –628 genotype and ALL, possibly interacting with high tungsten or arsenic levels, warrants further investigation. We are studying the prevalence of these variants in a larger population of children with ALL. We assume that there was no major change in the prevalence of alleles or exposure in the Churchill County population in the years preceding this investigation. We cannot rule out the possibility that exposures not accounted for in this investigation might have been associated with cases of leukemia either in conjunction with the documented exposures or unrelated to them; however, the findings of this study and those of Rubin et al. (2007) do not support an association of the known exposures with the temporal increase in the incidence of leukemia in Churchill County, Nevada.

## Figures and Tables

**Table 1:** Eleven cases of ALL in Churchill County, Nevada, by age group, diagnosis, WBC count, and cytogenetic studies.

Age group at diagnosis (years)	Diagnosis	WBC count	Cytogenetic results
12–20	Precursor B-cell ALL	63,700	No marrow stored
12–20	T-cell ALL	84,000	Metaphase cells show male karyotype with no clonal abnormality reported
2–10	Precursor B-cell ALL	6,800	Metaphase cells show normal male karyotype on GTG banding
2–10	Precursor B-cell ALL	216,500	Cells did not divide for metaphase analysis
2–10	Precursor B-cell ALL	3,900	Metaphase cells show male karyotype with no clonal abnormality reported
2–10	Precursor B-cell ALL	2,400	Metaphase cells show normal male karyotype
2–10	Precursor B-cell ALL	7,200	Abnormal karyotype consistent with ALL. T (12; 21) TEL-AML1 positive; MLL negative; E2A-PBX negative, BCR-ABL negative. Chromosome 12p aberration in association with chromosome 13q aberration
2–10	Precursor B-cell ALL	9,800	Metaphase cells show male karyotype with no clonal abnormality reported
2–10	Precursor B-cell ALL	2,600	Metaphase cells show abnormal female karyotype. Hyperdiploid clone in ~75% of cells
2–10	Precursor B-cell ALL	Pancytopenia	TEL-AML1 positive; MLL negative; E22A-PBX-1 negative; BCR-ABL negative
2–10	Precursor B-cell ALL	Not obtained	Not obtained

WBC, white blood cell.

Note: When translocations are not reported, neither the reverse-transcriptase polymerase chain reaction nor fluorescence *in situ* hybridization was done.

**Table 2:** Coding/regulatory region polymorphism discovery results for aldehyde oxidase, sulfate oxidase, and xanthine dehydrogenase.

					Predicted protein phenotype	
Polymorphism^a^	Gene position	Amino acid change	Minor allele case group	Frequencies comparison group^b^	PolyPhen^c^	SIFT^d^
*AOX1* 37630T→A	Exon 17	I598N	0.050 (20)	0 (42)	Probably damaging	Not tolerated (0.02)
*AOX1* 67888A→G	Exon 26	L957R	0.050 (20)	0 (48)	Benign	Tolerated (0.16)
*AOX1* 78499A→G	Exon 30	N1135S	0.091 (22)	0.119 (42)	Benign	Tolerated (1.00)
*AOX1* 86558A→G	Exon 34	H1297R	0.167 (12)	0.029 (34)	Benign	Tolerated (0.22)
*AOX1* 87950T→G	3′ UTR	NA	0.063 (16)	0.053 (38)	NA	NA
*AOX1* 88099G→A	3′ UTR	NA	0.056 (18)	0.043 (46)	NA	NA
*AOX1* 88169→delAT	3′ UTR	NA	0.556 (18)	0.413 (46)	NA	NA
*AOX1* 88248G→T	3′ UTR	NA	0.056 (18)	0.048 (42)	NA	NA
*SUOX–*628G→A	5′ UTR	NA	0.773 (22)	0.396 (48)	NA	NA
*SUOX –*619G→A	5′ UTR	NA	0.071 (14)	0 (46)	NA	NA
*SUOX –*586T→A	5′ UTR	NA	0.357 (14)	0.435 (46)	NA	NA
*SUOX –*429T→C	5′ UTR	NA	0 (14)	0.022 (46)	NA	NA
*SUOX –*317G→A	5′ UTR	NA	0 (16)	0.018 (40)	NA	NA
*SUOX* 1642G→A	Exon 6	E159L	0.056 (18)	0 (42)	Benign	Tolerated (0.15)
*SOUX* 1796C→T	Exon 6	P210L	0 (18)	0.024 (42)	Probably damaging	Not tolerated (0.00)
*XDH* 26391G→A	Exon 7	G172R	0 (20)	0.045 (44)	Benign	Not tolerated (0.05)
*XDH* 42390T→A	Exon 17	N602L	0 (14)	0.029 (34)	Probably damaging	Not tolerated (0.02)
*XDH* 42404G→A	Exon 17	R607Q	0 (14)	0.029 (34)	Benign	Not tolerated (0.04)
*XDH* 44269A→G	Exon 18	I646V	0.046 (22)	0 (48)	Benign	Tolerated (0.64)
*XDH* 75118A→T	Exon 34	I1238F	0 (18)	0.022 (46)	Benign	Tolerated (0.56)

NA, not applicable.

a Genomic DNA position coordinates are relative to Start ATG codon for each gene. Variations present in NCBI dbSNP database (http://www.ncbi.nlm.nih.gov/SNP/): *AOX1* 86558A→G (rs3731722), AOX1 87950T→G (rs1050884), *AOX1* 88099G→A (rs1050887), *AOX1* 88169A→delAT (rs34427902), *SUOX* –628G→A (rs7297662), *SUOX* –586T→A (rs773126), *SUOX* –317G→A(rs7963590), XDH 44269A→G (rs17323225).

b The number of sequenced chromosomes from the 11 case children and the 24 matched comparison children is in parentheses.

c PolyPhen prediction of functional effect of human nsSNPs ( http://genetics.bwh.harvard.edu/pph/).

d SIFT values < 0.05 are predicted to be deleterious.

**Table 3:** Gene characteristics and minor allele frequencies for polymorphisms (excluding *AOX1*, *SUOX*, and *XDH*).

		Minor allele frequency^a^	
Polymorphism	Amino acid change	Case group	Comparison group
*GSTP*1 313A→G	Ile→Val	0.25 (20)	0.36 (84)
*MBL2* codon 54A→G	Asp→Gly	0.23 (22)	0.21 (84)
*ABCB1* 3435C→T	Synonymous	0.35 (20)	0.52 (84)
*MTHFR* 677C→T	Ala→Val	0.41 (22)	0.40 (84)
*NQO1* 609C→T	Pro→Ser	0.20 (20)	0.25 (84)
*SLC19A1* 80A→G	His→Arg	0.45 (22)	0.48 (84)
*SHMT1* 1420C→T	Leu→Phe	0.36 (22)	0.21 (84)
*TPMT* 460G→A	Ala→Thr	0.00 (22)	0.06 (84)
*TPMT* 719A→G	Tyr→Cys	0.00 (22)	0.06 (84)
*VDR* start codon FokI		0.40 (20)	0.42 (84)
*TYMS* 28-bp repeat		0.41 (22)	0.43 (84)

a The number of sequenced chromosomes from the 11 case children and the 42 matched comparison children is in parentheses. Variations present in NCBI dbSNP database: *TPMT* 460G→A (rs1800460) and 719A→G (rs28933403), *SLC19A1* 80A→G (rs1051266), *SHMT1* 1420C→T (rs1979277), *GSTP1* 313A→G (rs1695), *NQO1* 609C→T (rs1800566), *MBL2* codon 54A→G (rs1800450), *ABCB1* 3435C→T (rs1045642), *MTHFR* 677C→T (rs1801133), and the *VDR* start site polymorphism (rs2228570).

**Table 4:** Univariate matched conditional logistic regression analyses for SNP loci in *AOXI*, *SUOX*, and *XDH*.

		95% CI^b^		
Polymorphism	Estimate^a^	Lower	Upper	*p*-Value^c^
*AOX1* 37630T →A	∞	0.05	∞	0.17
*AOX1* 67888A →G	∞	0.05	∞	0.17
*AOX1* 78499A →G	0.43	0.01	6.15	0.46
*AOX1* 86558A →G	∞	0.23	∞	0.17
*AOX1* 87950T →G	1.0	0.02	19.2	0.78
*AOX1* 88099G →A	1.14	0.02	22.1	0.78
*AOX1* 88196A →delAT	1.79	0.38	11.0	0.42
*AOX1* 88248G →T	1.14	0.02	22.1	0.78
*SUOX –*628G →A	6.81	1.28	257	0.007
*SUOX –*619G →A	∞	0.05	∞	0.17
*SUOX –*586T →A	0.74	0.13	2.99	0.66
*SUOX –*429T →C	0	0	78.0	0.67
*SUOX –*317G →A	—d	—d	—d	—d
*SUOX* 1642G →A	∞	0.03	∞	0.75
*SUOX* 1796C →T	0	0	117	0.63
*XDH* 26391G →A	0	0	13.1	0.33
*XDH* 42390T →A	0	0	78.0	0.67
*XDH* 42404G →A	0	0	78.0	0.67
*XDH* 44269A →G	∞	0.05	∞	0.17
*XDH* 75118A →T	0	0	117	0.63

a Point estimate of odds ratio of each copy of the minor allele on the odds of disease, obtained using matched conditional logistic regression. Values of ∞ (0) correspond to loci where the case (comparison) children had at least as many copies of the minor allele as the comparison (case) children in every informative stratum.

b Exact 95% CI values.

c Mid-P corrected *p*-value for the score test.

d For the SUOX –317G→A locus, there were no informative matched sets (i.e., no instances in which a case and at least one matched control had measured genotypes that were different).

**Table 5:** Univariate matched conditional logistic regression analyses for loci for all genes except *AOX1*, SUOX and XDH.

		95% CI^b^		
Polymorphism	Estimate^a^	Lower	Upper	*p*-Value^c^
*GSTP1* 313A→G	0.56	0.15	1.68	0.30
*MBL2* codon 54A→G	1.11	0.28	3.77	0.89
*ABCB1* 3435C→T	0.43	0.11	1.40	0.16
*MTHFR* 677C→T	1.05	0.35	3.31	0.90
*NQO1* 609C→T	0.75	0.17	2.63	0.68
*SLC19A1* 80A→G	0.91	0.30	2.71	0.90
*SHMT1* 1420C→T	1.95	0.64	6.87	0.17
*TPMT* 460G→A	0	0	4.36	0.43
*TPMT* 719A→G	0	0	4.36	0.43
*VDR* start codon FokI	0.93	0.26	2.97	0.90
*TYMS* 28-bp repeat	0.95	0.24	3.25	0.89

a Point estimate of odds ratio of each copy of the minor allele on the odds of disease, obtained using matched conditional logistic regression. Values of 0 correspond to loci where the comparison children had at least as many copies of the minor allele as the case children in every informative stratum.

b Exact 95% CI values.

c Mid-P corrected *p*-value for the score test.

**Table 6:** Transmission disequilibrium analysis.

	*p*-Value		Intact trios		Children with only one available parent (dyads)	
	Standard TDT^a^	1-TDT^b^	B^c^	C^d^	D^e^	E^f^
*GSTP1* 313A→G	1.0	0.65	2	2	0	1
*MBL2* codon 54A→G	0.71	0.71	3	4	0	0
*ABCB1* 3435C→T	0.26	0.26	2	5	0	0
*MTHFR* 677C→T	0.32	0.21	6	3	1	0
*NQO1* 609C→T	0.65	0.65	2	3	0	0
*SLC19A1* 80A→G	0.26	0.74	5	2	0	2
*SHMT1* 1420C→T	0.48	0.74	3	5	1	0
*TPMT* 460G→A	0.32	0.32	0	1	0	0
*TPMT* 719A→G	0.16	0.16	0	2	0	0
*VDR* start codon FokI	0.26	0.48	5	2	0	1
*TYMS* 28-bp repeat	0.48	0.21	3	5	0	2

a Standard TDT is (B − C)^2^/(B + C).

b 1-TDT including data from trios and dyads is (B + D − C − E)^2^/(B + D + C + E)*.

c Number of times a heterozygous parent transmitted the minor allele.

d Number of times a heterozygous parent transmitted the major allele.

e Number of times the case child had more minor alleles than the available parent.

f Number of times the case child had fewer minor alleles than the available parent.

**Table 7:** Hapotype analysis for common SNP loci in *AOX1*^a^ and *SUOX*^b^.

		β (*p*-value) for model		
Haplotpye	Frequency	Co-dominant	Dominant	Recessive
*AOX1*^a^				
000000 (A–A–T–G–A–G)	0.38	−0.59 (0.25)	−0.35 (0.74)	−∞ (0.02)
000010 (A–A–T–G–delA–G)	0.46	0.64 (0.20)	1.50 (0.09)	0.21 (0.82)
011101 (A–G–G–A–A–T)	0.05	0.16 (0.09)	0.20 (0.88)	−∞ (0.39)
100000 (G–A–T–G–A–G)	0.09	−1.94 (0.17)	–2.10 (0.18)	− ∞ (0.19)
110000 (G–G–T–G–A–G)	0.02	—b	—b	—b
*SUOX*				
00 (G–T)^c^	0.29	−1.42 (0.05)	−1.50 (0.11)	−∞ (0.05)
01 (G–A)	0.19	−1.56 (0.07)	−1.67 (0.10)	−∞ (0.12)
10 (A–T)	0.29	1.63 (0.02)	2.61 (0.01)	1.61 (0.32)
11 (A–A)	0.23	0.80 (0.23)	1.24 (0.18)	−0.93 (0.86)

a *AOX1* alleles with minor allele frequency of > 0.05 create a six maker haplotype of 78499A→G, 86558A→G, 87950T→G, 88099G→A, 88169A→delA and 88248G→T.

b Analysis not performed because the haplotype was too infrequent.

c *SUOX* alleles with minor allele frequency of > 0.05 form a two marker haplotype of –628G→A and –586T→A.
